# Skeletal muscle mass and sarcopenia can be determined with 1.5-T and 3-T neck MRI scans, in the event that no neck CT scan is performed

**DOI:** 10.1007/s00330-020-07440-1

**Published:** 2020-11-21

**Authors:** Aniek T. Zwart, Jan-Niklas Becker, Maria J. Lamers, Rudi A. J. O. Dierckx, Geertruida H. de Bock, Gyorgy B. Halmos, Anouk van der Hoorn

**Affiliations:** 1 Department of Epidemiology, University Medical Center Groningen, 30.001, Hanzeplein 1, 9700 RB Groningen, The Netherlands; 2 Department of Radiology, University Medical Center Groningen, Groningen, The Netherlands; 3 Department of Otolaryngology and Head and Neck Surgery, University Medical Center Groningen, Groningen, The Netherlands

**Keywords:** Sarcopenia, Head and neck neoplasms, Muscle, skeletal, Tomography, X-ray computed, Magnetic resonance imaging

## Abstract

**Objectives:**

Cross-sectional area (CSA) measurements of the neck musculature at the level of third cervical vertebra (C3) on CT scans are used to diagnose radiological sarcopenia, which is related to multiple adverse outcomes in head and neck cancer (HNC) patients. Alternatively, these assessments are performed with neck MRI, which has not been validated so far. For that, the objective was to evaluate whether skeletal muscle mass and sarcopenia can be assessed on neck MRI scans.

**Methods:**

HNC patients were included between November 2014 and November 2018 from a prospective data-biobank. CSAs of the neck musculature at the C3 level were measured on CT (*n* = 125) and MRI neck scans (*n* = 92 on 1.5-T, *n* = 33 on 3-T). Measurements were converted into skeletal muscle index (SMI), and sarcopenia was defined (SMI < 43.2 cm^2^/m^2^). Pearson correlation coefficients, Bland–Altman plots, McNemar test, Cohen’s kappa coefficients, and interclass correlation coefficients (ICCs) were estimated.

**Results:**

CT and MRI correlated highly on CSA and SMI (*r* = 0.958–0.998, *p* < 0.001). The Bland–Altman plots showed a nihil mean ΔSMI (− 0.13–0.44 cm^2^/m^2^). There was no significant difference between CT and MRI in diagnosing sarcopenia (McNemar, *p* = 0.5–1.0). Agreement on sarcopenia diagnosis was good with *κ* = 0.956–0.978 and *κ* = 0.870–0.933, for 1.5-T and 3-T respectively. Observer ICCs in MRI were excellent. In general, T2-weighted images had the best correlation and agreement with CT.

**Conclusions:**

Skeletal muscle mass and sarcopenia can interchangeably be assessed on CT and 1.5-T and 3-T MRI neck scans. This allows future clinical outcome assessment during treatment irrespective of used modality.

**Key Points:**

*• Screening for low amount of skeletal muscle mass is usually measured on neck CT scans and is highly clinical relevant as it is related to multiple adverse outcomes in head and neck cancer patients.*

*• We found that skeletal muscle mass and sarcopenia determined on CT and 1.5-T and 3-T MRI neck scans at the C3 level can be used interchangeably.*

*• When CT imaging of the neck is missing for skeletal muscle mass analysis, patients can be assessed with 1.5-T or 3-T neck MRIs.*

**Supplementary Information:**

The online version contains supplementary material available at 10.1007/s00330-020-07440-1.

## Introduction

Head and neck cancers have a great impact as combined they are the sixth most common cancer in Europe, with more than 250,000 cases and 63,500 deaths annually [1]. In The Netherlands, incidence of patients with head and neck cancer has increased by 47% between 1989 and 2019 [2]. Sarcopenia, or low skeletal muscle mass, is defined by the European Working Group on Sarcopenia in Older People (EWGSOP) as a progressive and generalized skeletal muscle disorder that is associated with increased likelihood of adverse outcomes [3]. Sarcopenia in head and neck cancer patients represents an important population burden with a prevalence of 6.6–70.9% and is related with frailty, post-operative complications, chemotherapy dose-limiting toxicity, and decreased overall survival and relapse-free survival [4–6]. Previous studies have indicated that females are more prone to sarcopenia than males [4, 7].

According to the EWGSOP, presence of low skeletal muscle mass (SMM) confirms the diagnosis of sarcopenia [3]. Single-slice assessment of SMM on neck CT scans at the level of the third cervical vertebra (C3) has recently been validated by Swartz et al [8] and could therefore be a breakthrough for a novel radiological biomarker in head and neck cancer patients. However, certain patients with head and neck cancer subtypes will not undergo CT imaging of the neck, but instead undergo imaging with the use of magnetic resonance imaging (MRI). This is especially the case not only for nasopharyngeal and sinonasal cancer, but also for other malignancies situated cranial to the hyoid bone [9–12], and in patients with iodinated contrast allergies.

Previous studies indicated that cross-sectional area (CSA) measurements on CT and MRI scans can be used interchangeably for muscles of the limbs [13], thigh [14], paraspinal skeletal back muscles [15, 16], and abdomen [17]. A recently published retrospective study indicates that CSA measurements of the neck musculature at the C3 level, made on CT and MRI scans, can also be used interchangeably [18], and additionally CSA measurements made on CT scans at the level of C3 are reproducible [19]. However, it has not yet been clarified if CSA measurements at the level of C3 are comparable for all MRI sequences and field strengths in relation to CT. It is also unknown if CSA measurements of the neck musculature on MRI at the C3 level are robust in context of reproducibility and repeatability. We hypothesize that SMM measurements on CT and MRI neck scans at the level of C3 are equivalent. Hence, our aim is to analyze the agreement and correlation of CSA measurements made on CT and various MRI neck scans made with different sequences and flied strengths at the level of C3.

## Materials and methods

Data was derived from OncoLifeS, a large oncological data-biobank [20]. Patients were prospectively included after written informed consent, and imaging data was retrospectively analyzed. The Medical Ethical Committee of the University Medical Centre of Groningen approved the data-biobank and the use of the data.

### Patients and study design

**Fig. 1:**
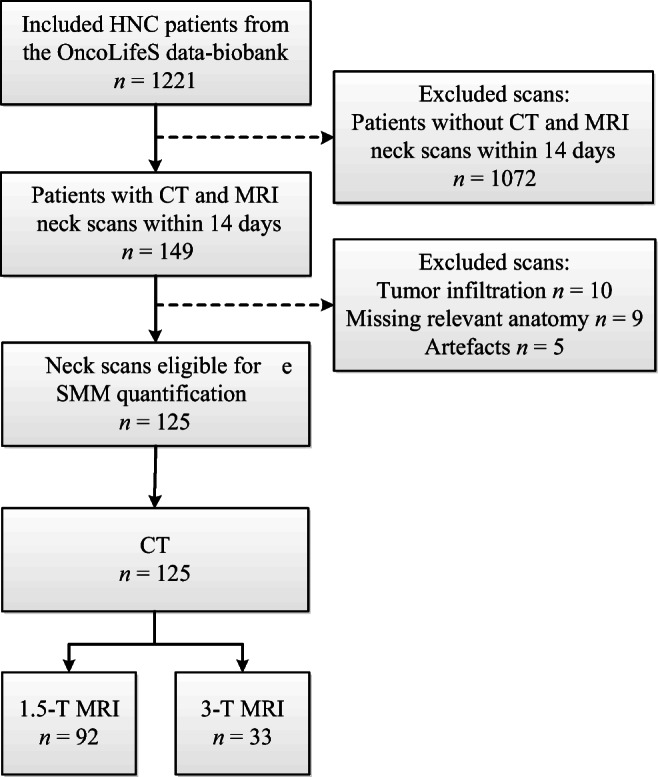
Flowchart of included and excluded patients. *HNC* head and neck cancer, *MRI* magnetic resonance imaging, *CT* computed tomography, *SMM* Skeletal muscle mass

Between November 2014 and November 2018, 1221 consecutive patients diagnosed with stage I–IV squamous cell carcinoma of the oral cavity, larynx, oropharynx, hypopharynx, and nasopharynx in the University Medical Centre of Groningen gave their informed consent in using their data in OncoLifeS (Fig. 1). In the presented analyses, patients were included with a maximum interval of 14 days between CT and MRI scans, to minimize time-dependent pathophysiological changes (*n* = 149). Excluded were scans with lymph node invasion into relevant muscles (*n* = 10), scans not capturing the relevant anatomy (*n* = 9), or scans with motion artefacts (*n* = 5). Total sample size was 125 patients with a mean age of 63 (42–82) years. Mean age for males was 64 (42–82) years and mean age for females was 61 (42–81) years.

### Image acquisition

All pre-treatment scans were acquired for clinical purposes and performed on a Siemens Healthcare CT (Biograph64, SOMATOM Force, SOMATOM Open, SOMATOM Definition AS or SOMATOM Definition Flash) and MRI scanners (1.5-T Area or 3-T Prisma or Skyra). CT scans were performed with intravenous iodine contrast (*n* = 115) or without (*n* = 10). CT image preference was a soft tissue kernel nearest to 30 (30, *n* = 78; 40, *n* = 39; 26, *n* = 8) with a slice thickness of 2.00 mm (2.00 mm, *n* = 107; 1.00 mm, *n* = 18).

Available sequences on 1.5-T MRI were T1 without contrast (repetition time/echo time (TR/TE) 2210–2780/55 ms, flip angle 150°, matrix 256 × 256, slice thickness 3 mm, and spacing between slices 3.6 mm), T1 vibe (volumetric interpolated breath-hold examination) with fat suppression and gadolinium 10–22 ml (TR/TE 5.04/2.34 ms, flip angle 10°, matrix 224 × 224, slice thickness 0.9 mm, 3D acquisition), and T2 (TR/TE 5990–8940/81 ms, flip angle 129–149°, matrix 320 × 320, slice thickness 3 mm, spacing between slices 3.6 mm).

For 3-T, the sequences used were T1 turbo spin echo (TSE) without contrast and T1 TSE with gadolinium 11–21 ml (both TR/TE 893–1020/11 ms, flip angle 139–160°, matrix 640 × 640, slice thickness 3 mm, spacing between slices 3.6 mm), T1 vibe 3D-DIXON with fat suppression and gadolinium 11–21 ml (TR/TE 4.49–5.5/2.46 ms, flip angle 9°, matrix 256–264 × 256–264, slice thickness 0.9 mm), and T2 TSE DIXON (TR/TE 5460–6880/77–97 ms, flip angle 120–126°, matrix 640 × 640, slice thickness 3 mm, spacing between slices 3.6 mm).

### Skeletal muscle image analysis

**Fig. 2:**
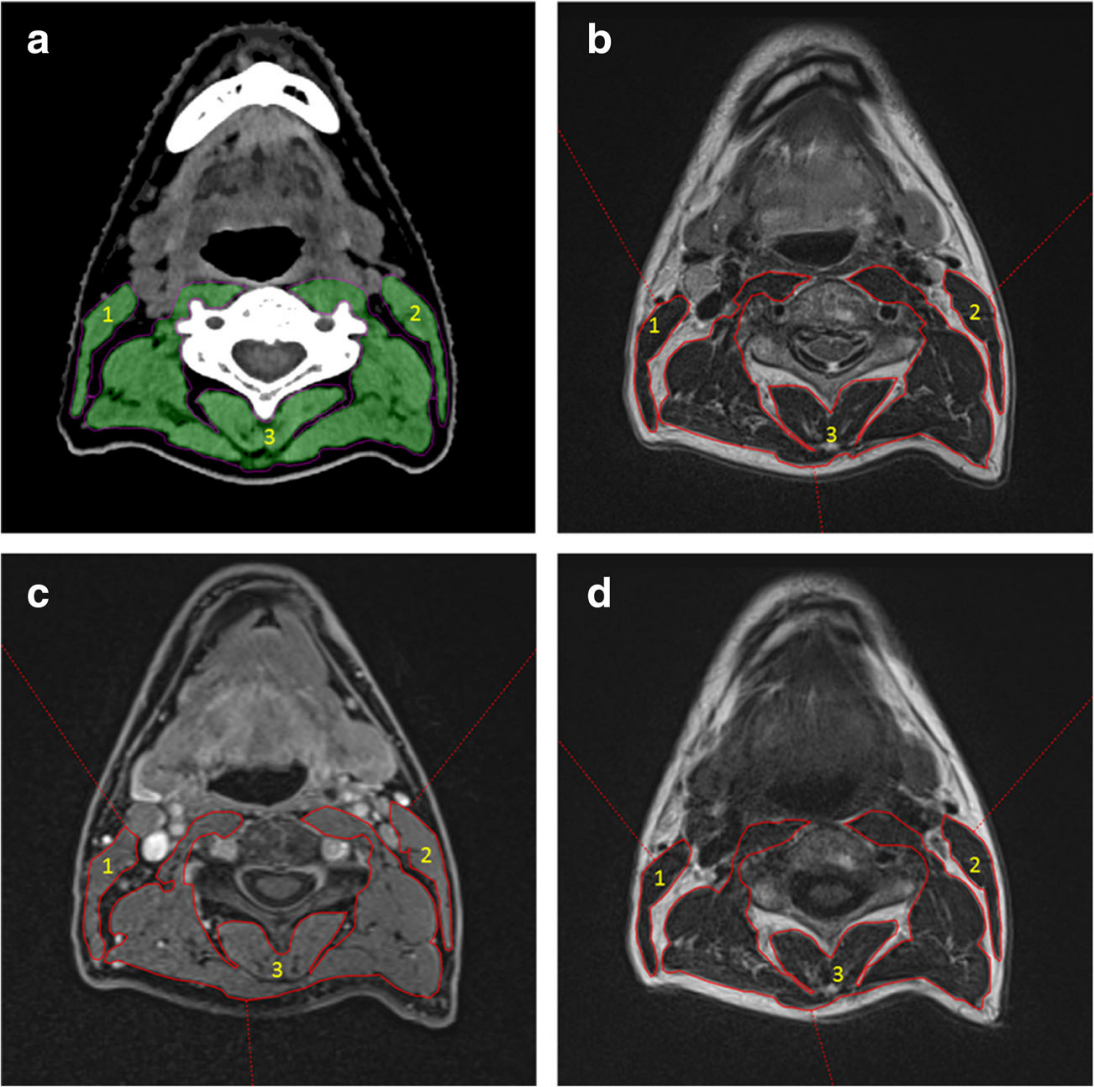
Skeletal muscle measurements at the level of C3 on axial neck CT and MRI images. (1) CSA of the right sternocleidomastoids muscle, (2) CSA of the left sternocleidomastoids muscle, and (3) CSA of the paravertebral muscles. **a** Acquired using CT. The green area represents the delineated muscle area which corresponds with the preset Hounsfield units range of − 29 until 150. Other densities are automatically excluded from the measured area. **b–d** Slices acquired using 1.5-T MRI. The before-mentioned muscles are delineated red with (**b**) T2 without fat suppression, (**c**) T1 with contrast and fat suppression, and (**d**) being T1 sequence without contrast and without fat suppression. *C3* third cervical vertebra, *CT* computed tomography, *MRI* magnetic resonance imaging, *CSA* cross-sectional area

SMM quantification was conducted with the Aquarius workstation iNtuition edition program (v.4.4.13.P6, TeraRecon, Inc.). Slice selection was performed according the validated procedure of Swartz et al [8]. However, MRI scans had a relatively large pitch and slice thickness. If a fully closed arch could not be identified, the most caudal slice was chosen where the posterior arch was nearest to a closed arch (Fig. 2). Interpolation between images was utilized, and angulation was prohibited to ensure reproducibility.

CT measurements were performed as described earlier [4] (Fig. 2a). Delineation made on the MRI sequences was done manually, eyeballing the relevant structures including the paravertebral muscles and both sternocleidomastoid muscles. Total CSA on MRI corresponded with the true delineated area.

### Observer reliability

All measurements were performed by JNB (obs. 1; medical student) after an extensive training of 2 weeks under the supervision of ATZ and AvdH (board-certified neuro/head–neck radiologist with 3 years of experience with these specific measurements). Interobserver reliability was analyzed with intraclass correlation coefficients. Interobserver analysis was performed in a randomly reselected sample (*n* = 25) on 1.5-T neck MRI scans by ATZ (obs. 2; PhD student with a background in medicine and as a radiologic technician, with 3 years of experience with the used acquisition program) and MJL (obs. 3; with 7 years of experience as a board-certified neuro/head–neck radiologist, without specific training needed). Level of slice selection was also analyzed per observer and measurement data were blinded for the observers. Furthermore, intraobserver analysis was done with a time interval of > 2 weeks between the first and the second CSA measurements (*).

### Sarcopenia diagnosis

Skeletal muscle index (SMI, cm^2^/m^2^) at the level of L3 was determined, which is considered a surrogate marker for the total body SMM [21]. First, CSA at C3 (cm^2^) was converted to CSA at L3 (cm^2^) according the algorithm of Swartz et al [8] (see Eq. 1). Second, calculated CSA at L3 was furthermore adjusted for patient height (m^2^) resulting in SMI (see Eq. 2). The outcome, or SMM status, was presented continuously with SMI, and dichotomously as (non-)sarcopenic based on previously published SMI cut-off value (< 43.2 cm^2^/m^2^) [7].

1 $$ {\displaystyle \begin{array}{c} CSA\  at\ L3\ \left({cm}^2\right)=27.304+1.363\ast CSA\  at\ C3\ \left({cm}^2\right)-0.671\ast Age\ (years)\\ {}+0.640\ast Weight\ (kg)+26.442\ast Sex\ \left(1= Female,2= Male\right)\end{array}} $$

2 $$ SMI\ \left({cm}^2/{m}^2\right)= CSA\  at\ L3\ \left({cm}^2\right)/ Height\ \left({m}^2\right) $$

### Statistical analysis

Continuous data was analyzed for normality with the Shapiro–Wilk test (normality: *α* > 0.05) and Q-Q plots. The baseline characteristics of the patient cohort were summarized. Differences of baseline characteristics between patients in the 1.5-T group and those in the 3-T group were analyzed with independent *t* tests and Pearson’s chi-square tests. CSA measurements made with CT and MRI were analyzed with Pearson’s correlation coefficient. CT was selected as the reference standard, as previously described algorithm was generated on neck CTs. Bland–Altman plots with 95% confidence intervals were created with mean SMI (CT + MRI/2) and ΔSMI (CT − MRI) to visualize agreement, possible biases, or outliers. A linear regression was performed and added to the Bland–Altman plot when significant. Interclass correlation coefficients (ICCs) were performed to analyze observer reliability. Data was furthermore stratified for gender, as gender differences were previously observed [4, 7]. To estimate clinical relevance, difference and agreement of sarcopenia diagnosis between CT and MRI were analyzed with the McNemar test and Cohen’s kappa coefficient (*κ*) respectively. A non-significant McNemar test corresponds with no difference in diagnosis between the two modalities [22], and a *κ* > 0.81 was considered perfect agreement [23]. Variables were statistically significant if *α* < 0.05. There was no missing data. SPSS version 23.0 was used for statistical analysis.

## Results

### Patient and disease characteristics

The intended sample size consisted of 125 head and neck cancer patients with pre-treatment CT and MRI scans (see Table 1 for baseline characteristics). The majority of the patient sample was male (72%), and the mean age at time of diagnosis was 63 (± 9) years. Most patients had oropharyngeal cancer (48%), followed by laryngeal (36%), hypopharyngeal (14%), oral (1%), and nasopharyngeal cancer (1%). Three quarters of patients had stage III–IV advanced disease. None of the baseline variables showed significant differences (*p* = 0.053–0.84) between 1.5-T and 3-T MRI scans. All variables (characteristics as well outcomes) were normally distributed (Shapiro–Wilk: *p* > 0.05).

**Table 1:** Characteristics of included patients

	1.5-T (*n =* 92)	3-T (*n =* 33)	*p* value
Sex			0.31^a^
Female	28 (30.4%)	7 (21.2%)	
Male	64 (69.6%)	26 (78.8%)	
Age (years)	62.7 (± 9.0)	64.4 (± 10.3)	0.37^b^
BMI (kg/m^2^)	25.0 (± 5.3)	25.2 (± 4.0)	0.82^b^
Tumour site			0.13^a^
Oropharynx	48 (52.2%)	12 (36.4%)	
Larynx	28 (30.4%)	17 (51.5%)	
Hypopharynx	15 (16.3%)	3 (9.1%)	
Oral cavity or nasopharynx	1 (1.1%)	1 (3.0%)	
T-classification*			0.63^b^
T1	12 (13.0%)	2 (6.1%)	
T2	23 (25.0%)	7 (21.2%)	
T3	31 (33.7%)	14 (42.4%)	
T4	26 (28.3%)	10 (30.3%)	
N-classification*			0.053^a^
N0	25 (37.2%)	15 (45.5%)	
N+	67 (62.8%)	18 (54.5%)	
Oncologic stage*			0.84^a^
I–II	2 (2.2%)	4 (12.1%)	
III	22 (23.9%)	9 (27.3%)	
IV	61 (66.3%)	22 (60.6%)	

Patients stratified according to MRI field strength 1.5-T and 3-T. Categorical data is given with percentage of total group size *n*. Continues data is given as mean with standard deviation. Significance *p* calculated by ^a^Pearson’s chi-square test and ^b^Student’s independent *t* test. *Staging confirmed with the 7th edition of the American Joint Committee on Cancer Manual

### Correlation and agreement between CT and MRI

Mean time between CT and MRI was 3.27 ± 3.42 days for 1.5-T scans and 1.94 ± 2.76 days for 3-T scans. The scores for CSAs and SMI as measured with CT and MRI for both 1.5-T and 3-T were highly correlated (*r* = 0.958–0.997, *p* < 0.001) (Table 2). Some minor differences were observed when analyzing the three delineated structures separately, as the paravertebral muscles scored relatively the highest correlation (*r* = 0.988–0.995) compared to the left of the right sternocleidomastoid muscle (*r* = 0.958–0.976 and *r* = 0.961–0.986 respectively). T2 DIXON sequence on the 3-T MRI scanner had marginally the highest correlation based on total CSA and SMI (*r* = 0.998).

**Table 2:** Correlation coefficients and sarcopenia categorization between CT and MRI sequences

	Total CSA	RSM	LSM	PM	SMI
1.5-T					
T1	0.987*	0.969*	0.966*	0.988*	0.997*
T1 vibe	0.989*	0.961*	0.958*	0.989*	0.997*
T2	0.988*	0.971*	0.959*	0.988*	0.997*
3-T					
T1	0.990*	0.986*	0.976*	0.991*	0.997*
T1 + contrast	0.989*	0.967*	0.970*	0.991*	0.997*
T1 DIXON	0.990*	0.980*	0.976*	0.990*	0.997*
T2 DIXON	0.994*	0.965*	0.962*	0.995*	0.998*

Comparing CT with 1.5-T (*n = 92*) and 3-T (*n = 33*) MRI sequences with Pearson’s correlation coefficients for area measurements and the skeletal muscle index. *CT* computed tomography, *MRI* magnetic resonance imaging, *CSA* cross-sectional area, *RSM* right sternocleidomastoid muscle, LSM left sternocleidomastoid muscle, PM paravertebral muscle, *SMI* skeletal muscle index, Sarc. sarcopenic. *Significant *p* value < 0.001

**Fig. 3:**
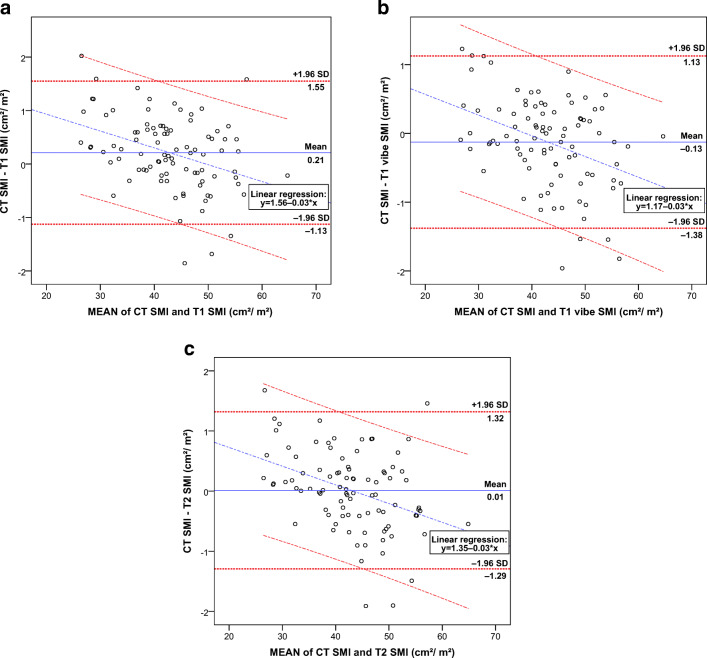
Bland–Altman plots with mean SMI and ΔSMI between CT and 1.5-T MRI. Boundaries with the 95% confidence interval (± 1.96 times the standard deviation) are given for the mean ΔSMI and linear regression analysis. For all patients with a 1.5-T MRI (*n* = 92) comparing CT SMI versus T1 SMI (**a**), CT SMI versus T1 vibe SMI (**b**), and CT SMI versus T2 SMI (**c**). *SMI* skeletal muscle index, *CT* computed tomography, *MRI* magnetic resonance imaging

When visualizing the agreement between the 1.5-T MRI scans and CT, a few outliers (*n* = 5–6) could be identified per sequence (Fig. 3). Noticeably, almost all outliers with the high mean SMI region were found under the 95% CI and vice versa. This trend was also illustrated when adding the regression line, which had a downward slope of − 0.03 showing a small proportional bias. Mean ΔSMI was almost zero for all sequences, with the lowest delta of 0.01 cm^2^/m^2^ for the CT and T2 sequence, and the highest delta of 0.21 cm^2^/m^2^ for the CT and T1 sequence.

Bland–Altman plots between CT and the four 3-T MRI sequences were very similar to the 1.5-T sequences (see Fig. 1 in supplementary results), with minimal mean ΔSMI. T2 DIXON was superior with the lowest mean ΔSMI of 0.17 cm^2^/m^2^.

### Observer agreement of CSA measurements in MRI

Analysis on slice selection (Table 3) gave similar results for T1 and T2; in 56–64% of the cases, the same slice was selected and the deviation was solely one slice. Selecting the same level was harder for T1 vibe, as 16–24% of the cases the same slice was selected, and more than half of the cases had a deviation of 2 or more slices.

**Table 3:** Inter- and intraobserver reliability and slice selection

Interobserver correlation with slice selection							Intraobserver correlation		
	CSA	Slice	Obs. 1	Obs. 2	Obs. 3	ICCs (95% CI)	First	Second	ICCs (95% CI)
T1	Total		38.95 (± 9.11)	39.40 (± 9.78)	39.11 (± 10.10)	0.996 (0.993–0.998)*	39.07 (± 9.00)	38.95 (± 9.11)	0.998 (0.994–0.999)*
	PM		32.59 (± 7.66)	32.86 (± 8.18)	32.64 (± 8.54)	0.996 (0.992–0.998)*	32.61 (± 7.54)	32.59 (± 7.66)	0.997 (0.993–0.999)*
	RSM		3.17 (± 0.85)	3.27 (± 0.92)	3.24 (± 0.91)	0.993 (0.986–0.997)*	3.20 (± 0.83)	3.17 (± 0.85)	0.992 (0.981–0.996)*
	LSM		3.19 (± 0.81)	3.27 (± 0.90)	3.22 (± 0.89)	0.994 (0.988–0.997)*	3.26 (± 0.83)	3.19 (± 0.81)	0.985 (0.958–0.994)*
		Same	Ref.	16 (64%)	16 (64%)				
		1	Ref.	9 (36%)	9 (36%)				
T1 vibe	Total		39.51 (± 9.16)	6 (24%)	4 (16%)	0.993 (0.983–0.997)*	39.72 (± 9.14)	39.51 (± 9.16)	0.997 (0.993–0.999)*
	PM		33.14 (± 7.67)	33.86 (± 8.88)	33.32 (± 8.58)	0.993 (0.987–0.997)*	33.16 (± 7.63)	33.14 (± 7.67)	0.998 (0.995–0.999)*
	RSM		3.19 (± 0.90)	3.57 (± 0.96)	3.27 (± 0.97)	0.973 (0.877–0.991)*	3.25 (± 0.87)	3.19 (± 0.90)	0.975 (0.944–0.989)*
	LSM		3.18 (± 0.81)	3.50 (± 0.96)	3.23 (± 0.90)	0.968 (0.905–0.987)*	3.31 (± 0.85)	3.18 (± 0.81)	0.961 (0.870–0.985)*
		Same	Ref.	6 (24%)	4 (16%)				
		1	Ref.	5 (20%)	8 (32%)				
		≥ 2	Ref.	14 (56%)	13 (52%)				
T2	Total		39.37 (± 9.17)	40.30 (± 9.94)	39.67 (± 9.94)	0.995 (0.990–0.998)*	39.51 (± 9.09)	39.37 (± 9.17)	0.998 (0.996–0.999)*
	PM		32.87 (± 7.67)	33.60 (± 8.40)	33.23 (± 8.43)	0.994 (0.989–0.997)*	32.95 (± 7.61)	38.89 (± 7.67)	0.998 (0.996–0.999)*
	RSM		3.25 (± 0.86)	3.39 (± 0.91)	3.24 (± 0.89)	0.991 (0.997–0.996)*	3.28 (± 0.85)	3.25 (± 0.86)	0.992 (0.982–0.996)*
	LSM		3.22 (± 0.85)	3.31 (± 0.86)	3.21 (± 0.85)	0.995 (0.987–0.998)*	3.28 (± 0.83)	3.22 (± 0.85)	0.986 (0.965–0.994)*
		Same	Ref.	16 (64%)	14 (56%)				
		1	Ref.	9 (36%)	11 (44%)				

ICCs for inter- and intraobserver analyses of skeletal muscle area measurements made by all three observers, and relative difference of slice selection between first observer and other observers *n = 25*. *CSA* cross-sectional area, *CI* confidence interval, *ICCs* intraclass correlation coefficients, *PM* paravertebral muscles, *RSM* right sternocleidomastoid muscle, *LSM* left sternocleidomastoid muscle. *Significant *p* value < 0.001

Excellent and significant intra- and interobserver reliability were achieved in the randomly reselected patients from the 1.5-T MRI group (Table 3). The intraobserver ICCs for total CSA and CSA of PM at the level of C3 were the highest (*r* = 0.997–0.998, *p* < 0.001), independently of sequence, and T1 has the best interobserver ICC (*r* = 0.996, *p* < 0.001). However, differences of ICCs between the three 1.5-T MRI sequences made were all marginal.

### Agreement in SMI in CT and MRI stratified for gender

**Fig. 4:**
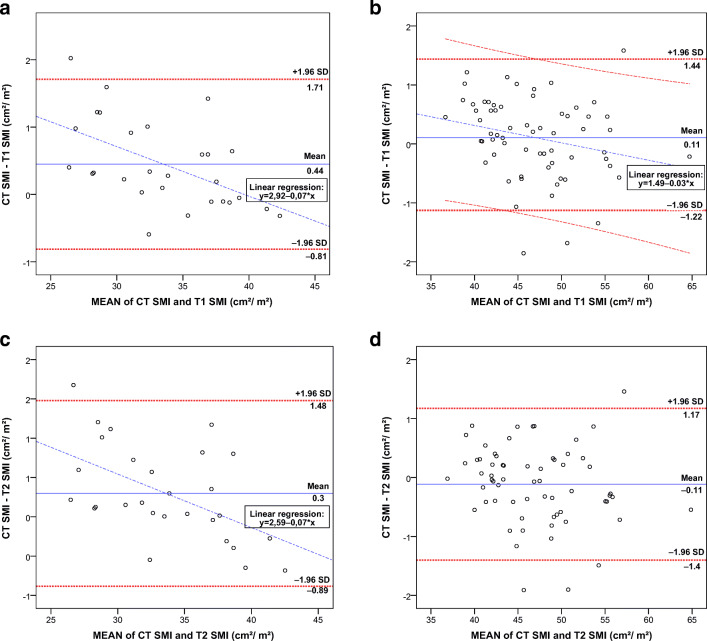
Bland–Altman plots of mean SMI and ΔSMI between CT and 1.5-T MRI stratified for gender. Boundaries with the 95% confidence interval (± 1.96 times the standard deviation) are given for the mean ΔSMI and linear regression analysis, except for **d**. For women (*n* = 28), CT SMI versus T1 SMI (**a**) and CT SMI versus T2 SMI (**c**); and for men (*n* = 64), CT SMI versus T1 SMI (**b**) and CT SMI versus T2 SMI (**d**). *SMI* skeletal muscle index, *CT* computed tomography, *MRI* magnetic resonance imaging

Subsequently, the mean ΔSMI between CT and the three 1.5-T sequences were stratified into men (*n* = 64) and women (*n* = 28) in Bland–Altman plots (Fig. 4, for T1 and T2). For men, the mean ΔSMI was 0.11 cm^2^/m^2^ for both T1 and T2 and 0.23 cm^2^/m^2^ for T1 vibe. Here, the regression line was significant for T1 vibe (*p* = 0.037) but not for T1 (*p* = 0.051) and T2 (*p* = 0.156).

For women, the mean ΔSMI was the closest to zero for T1 vibe with 0.1 cm^2^/m^2^ (0.4 cm^2^/m^2^ for T1 and 0.3 cm^2^/m^2^ for T2). For women, linear regressions were all significant (*p* < 0.001) and displayed a proportional bias with a downward slope of − 0.07, which was relatively higher than in men (− 0.03–0), but remained a minor difference.

### Effect of use of CT or MRI on presence of sarcopenia diagnosis

Categorization of sarcopenic patients with the different MRI field strengths compared to CT is shown in Table 4. With 1.5-T MRI sequences, one (+ 2%: T2) or two (+ 4%: T1) additional patients were categorized as sarcopenic (SMI < 43.2 cm^2^/m^2^), except for T1 vibe as one patient was not categorized as sarcopenic (− 2%). Agreement of CT-derived SMI was almost perfect for all 1.5-T sequences (*κ* ≥ 0.956).

**Table 4:** Sarcopenia categorization between CT and MRI

	SMI < 43.2 cm^2^/m^2^	SMI ≥ 43.2 cm^2^/m^2^	Cohen’s *κ* (95% CI)	McNemar
1.5-T				
CT SMI	*n =* 47	*n =* 45		*p* value
T1 SMI	49 (+ 4%)	43 (− 4%)	0.956 (0.897–1)*	0.500
T1 vibe SMI	46 (− 2%)	46 (+ 2%)	0.978 (0.936–1)*	1.000
T2 SMI	48 (+ 2%)	46 (− 2%)	0.978 (0.936–1)*	1.000
3-T				
CT SMI	*n =* 11	*n =* 22		
T1 SMI	13 (+ 18%)	20 (− 9%)	0.870 (0.725–1)*	0.500
T1 contrast SMI	13 (+ 18%)	20 (− 9%)	0.870 (0.725–1)*	0.500
T1 DIXON SMI	13 (+ 18%)	20 (− 9%)	0.870 (0.725–1)*	0.500
T2 DIXON SMI	12 (+ 9%)	21 (− 4%)	0.933 (0.827–1)*	1.000

Relative difference of sarcopenia diagnosis for both 1.5-T (*n = 92*) and 3-T (*n = 33*) *MRI* sequences respective to CT, with agreement and differences of sarcopenia categorization between the two modalities analyzed with Cohen’s kappa coefficient and McNemar test, respectively. *SMI* skeletal muscle index, *CI* confidence interval. *Significant *p* value < 0.001

One (+ 9%: T2 DIXON) or two (+ 18%: T1, T1 contrast, and T1 DIXON) additional categorized sarcopenic patients were also seen in measurements made on 3-T sequences. The three T1 sequences had therefore relatively a lower agreement (*κ* = 0.870) in contrast to T2 DIXON (*κ* = 0.933), nevertheless still both good. These differences were only found in men, as all women were identified as sarcopenic by all methods using the before mentioned cut-off value. The exact McNemar test using a binominal distribution showed no significance in sarcopenia categorization between CT and the examined MRI sequences (*p* ≥ 0.50).

## Discussion

This is the first study with CSA data on the level of C3 generated on MRI with different field strengths and sequences, in relation to CT. We found an excellent correlation between CSA measurements on CT and 1.5-T and 3-T MRI neck scans at the level of C3. Differences in SMI (mean ΔSMI) between CT and MRI were minimal with good agreement and less than 5% outliers outside the 95% CI. Never previously published observer analysis for MRI in this context showed that slice selection was the easiest for T1 and T2 sequences in the 1.5-T group, and CSA measurements are both reliable and reproducible, as we found excellent intra- and interobserver ICCs. Agreement of CT and MRI was practically the same for men and women. Equivalent diagnosis of sarcopenia was made with both modalities. However, preference could be made to utilize T2-weighted images as highest intra- and interobserver agreements, correlation, and agreements on Bland–Altman plots were achieved.

High correlations and agreement of CSA measurements of muscles on CT and MRI are universally found in previous articles [13–18]. We found, irrespective of the sequence or field strength, an excellent correlation for total CSA in the neck scans. Our correlation is higher than previous found for neck muscles at C3 [18], paraspinal back muscles [15], and abdominal muscles at L1 [17]. As Chargi et al [18] did not specify MRI acquisition parameters, it is hard to compare results. The study of Sinelnikov et al [17] using threshold-based region growing segmentation also differs from this study and therefore hampers a direct comparison. Nevertheless, they found the highest correlation and agreement in T2-weighted images [17], which is in line with our present study. 1.5-T and 3-T MRI also showed previous excellent agreement in a small animal study comparing histology and CSA measures of different muscles [24]. The found small proportional bias might be based on the different methods used for CT and MRI delineation of CSA, which is similar to other studies that compared manual and threshold-based techniques in CSA measurements [25, 26]. This finding was more emphasized in women probably due to relatively lower SMI ratios. Nevertheless, the agreement using the 95% CI identified only a few outliers in our study. Bril et al [19] also found a significantly excellent interobserver agreement in CSA measurements on neck CT scans at the level of C3. Similar to our study, the highest ICCs in MRI were found in CSA of paravertebral muscles and total CSA at C3, and lowest ICCs in CSA of sternocleidomastoid muscle independently. Our results exceeded the intra- and interobserver ICCs in the study of Sinelnikov et al and Khil et al on abdominal scans [16, 17], possibly due to different delineation methods used for MRI in their studies. This further indicates the robustness of our applied methods for CSA measurements in 1.5-T MRI, using semi-automated for CT and manual delineation for MRI to determine sarcopenic patients.

However, some limitations have to be mentioned. External validation of our findings could be considered the major limitation of this present study. This is due to missing cross-validation of the formula of Swartz et al to calculate CSA at the L3 level through CSA at the C3 level, and the lack of large-scale validated SMI cut-off values for sarcopenia detection in head and neck cancer patients [7, 8]. However, a recent published study found a strong and significant correlation between SMI determined at the C3 and L3 level [27]. Further research should therefore be focussed on cross-validation of the formula of Swartz et al and head and neck cancer patient–specific SMI cut-off values for determining sarcopenia in larger cohorts. We applied the gender independent SMI cut-off value of Wendrich et al as it has a very similar head and neck cancer population. In previous studies, the use of gender-dependent cut-off values was proposed [7, 28], but also seen as a difficult task as women have a smaller ratio within the head and neck cancer population. Furthermore, qualitative research with muscle CT radio-density should be validated, as recently muscle density was reported to be more associated with frailty in older adults with cancer, than skeletal muscle area [29]. CSA measurements should be validated on low-dose CT neck scans to ensure that all head and neck cancer patients can be screened for sarcopenia before, during, and after treatment. Our study has nevertheless multiple strengths. Firstly, the study was performed in a relatively large group of patients from a prospectively maintained data-biobank. Secondly, an excellent inter- and intraobserver agreement by three observers was demonstrated in the random reselected neck CT and MRI scans, proving that CSA measurements at the level of C3 are both reproducible and reliable. Thirdly, high grades can be given for the short time interval between CT and MRI minimizing the impact of pathophysiological muscle change to an absolute minimum. Furthermore, data was stratified for gender as previous studies emphasized that SMI differences exist between men and women.

In conclusion, CSA measurements on CT and 1.5-T and 3-T MRI neck scans at the C3 level can be used interchangeably. In the event no neck CT scan is performed, skeletal muscle mass and radiological sarcopenia can be determined with CSA measurements on neck MRI scans. This finding contributes to the construction of a clinical useful radiological biomarker for measuring radiological sarcopenia in head and neck cancer patients, which has previously been emphasized by others [18, 19].

## Supplementary Information

ESM 1 Supporting information Fig. 1. Bland-Altman plots with mean SMI and ΔSMI between CT and 3 Tesla MRI. Boundaries with the 95% confidence interval (±1.96 times the standard deviation) are given for the mean ΔSMI and linear regression analysis. For all patients of the 3 Tesla group (n=33) CT SMI vs. T1 SMI (**a**), CT SMI vs. T1with contrast SMI (**b**), CT SMI vs. T1 DIXON SMI (**c**) and CT SMI vs. T2 DIXON SMI. Abbreviations: *SMI* Skeletal Muscle Index, *CT* Computed Tomography, *MRI* Magnetic Resonance Imaging (PDF 27.2 kb)

## Abbreviations

C3: Third cervical vertebra
CSA: Cross-sectional area
HU: Hounsfield unit
L3: Third lumbar vertebra
LSM: Left sternocleidomastoid muscle
PM: Paravertebral muscles
RSM: Right sternocleidomastoid muscle
SMI: Skeletal muscle index
SMM: Skeletal muscle mass
