# Supplementary figures and images for: Skeletal muscle mass and sarcopenia can be determined with 1.5-T and 3-T neck MRI scans, in the event that no neck CT scan is performed
Source: Eur Radiol. 2020 Nov 21;31(6):4053–62. doi: 10.1007/s00330-020-07440-1 (PMC8128750; doi:10.1007/s00330-020-07440-1)

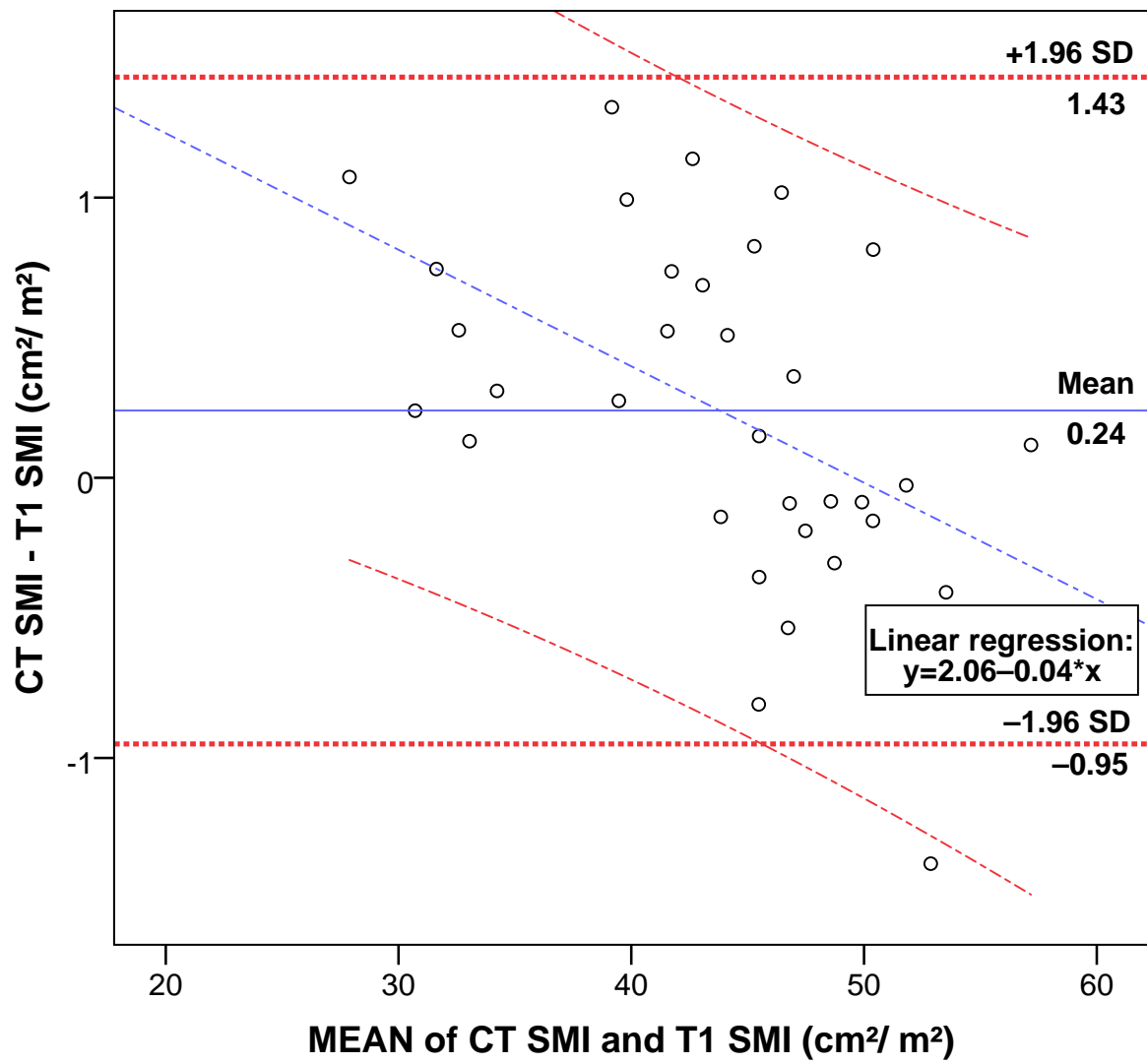

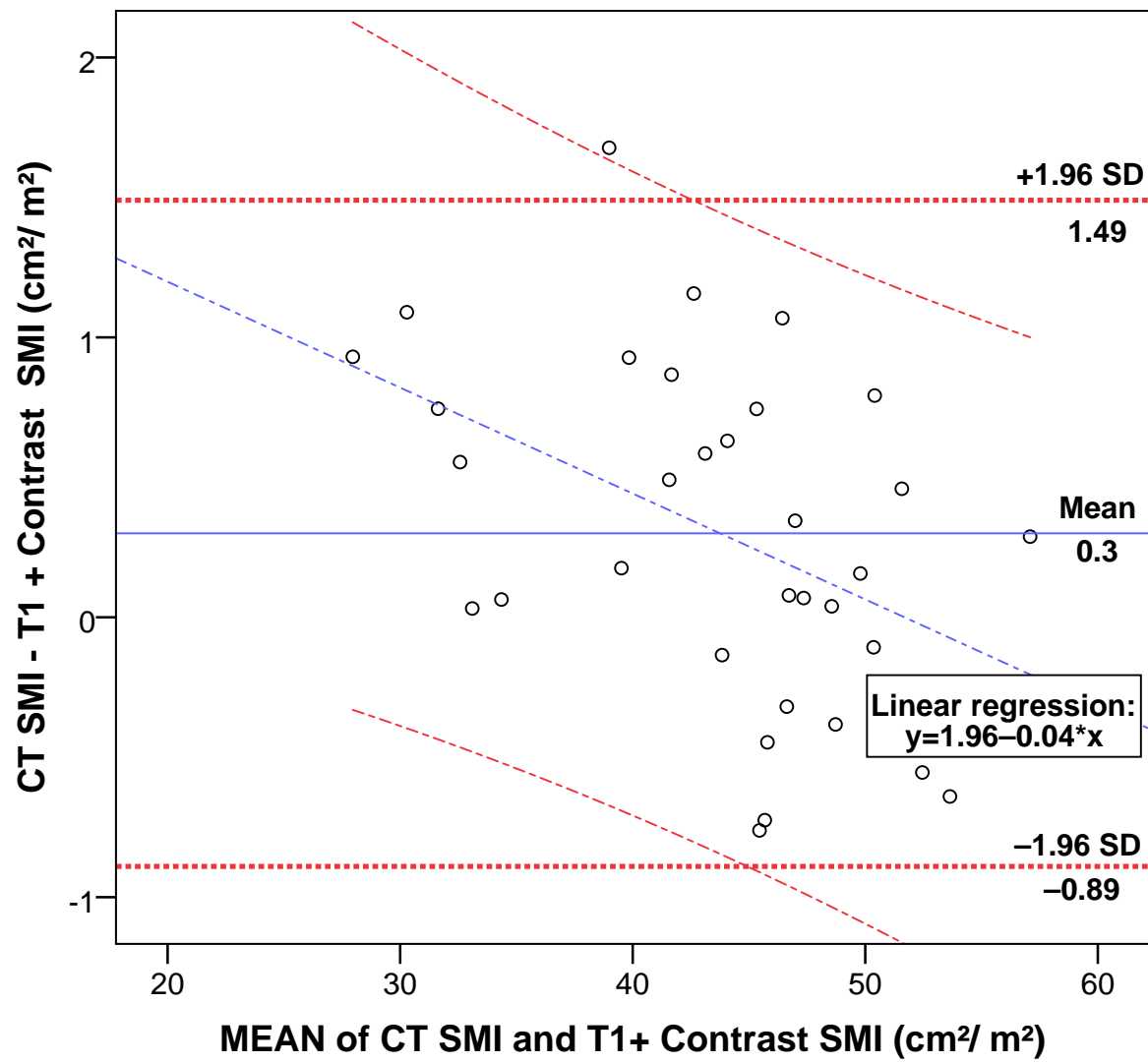

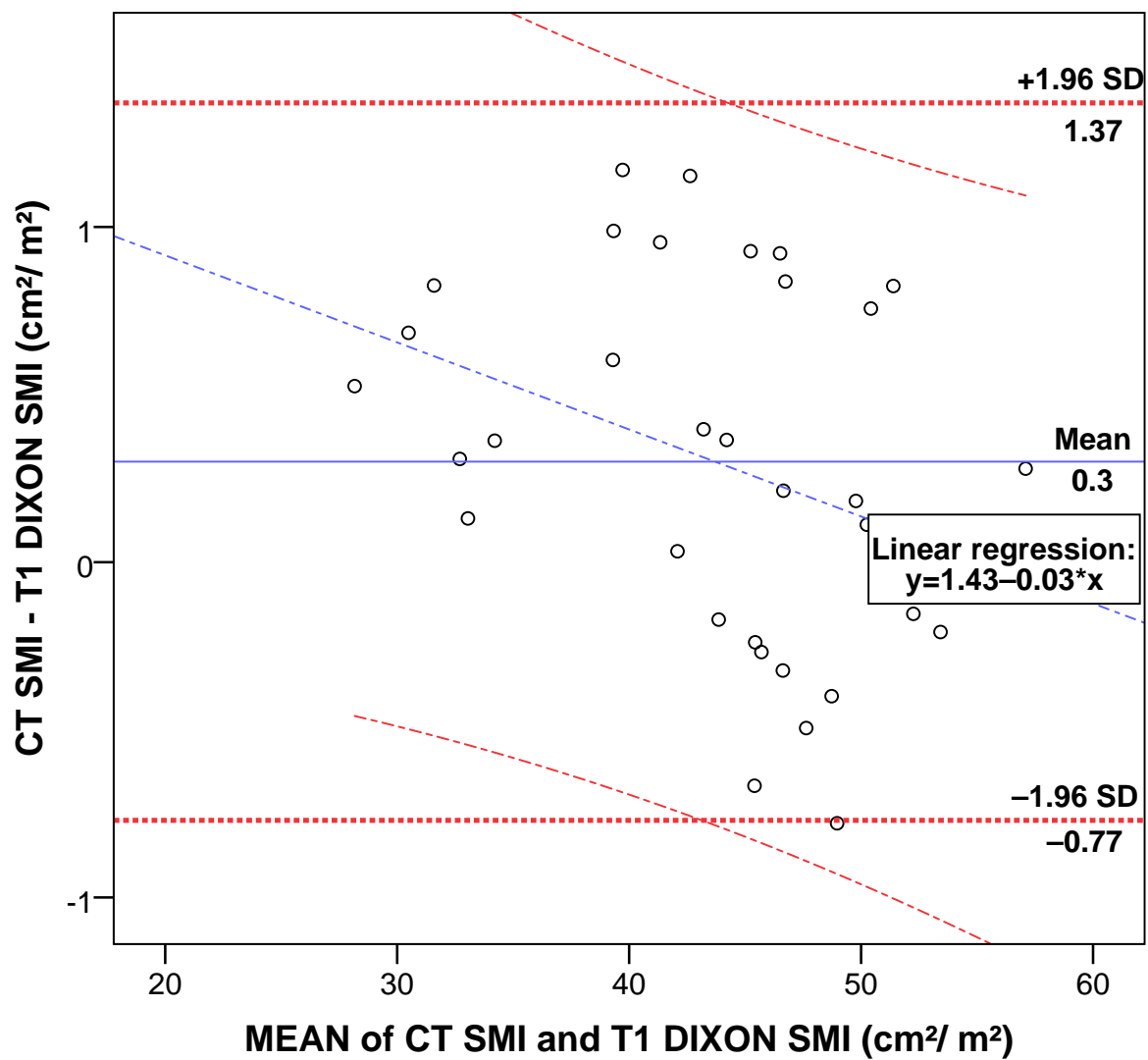

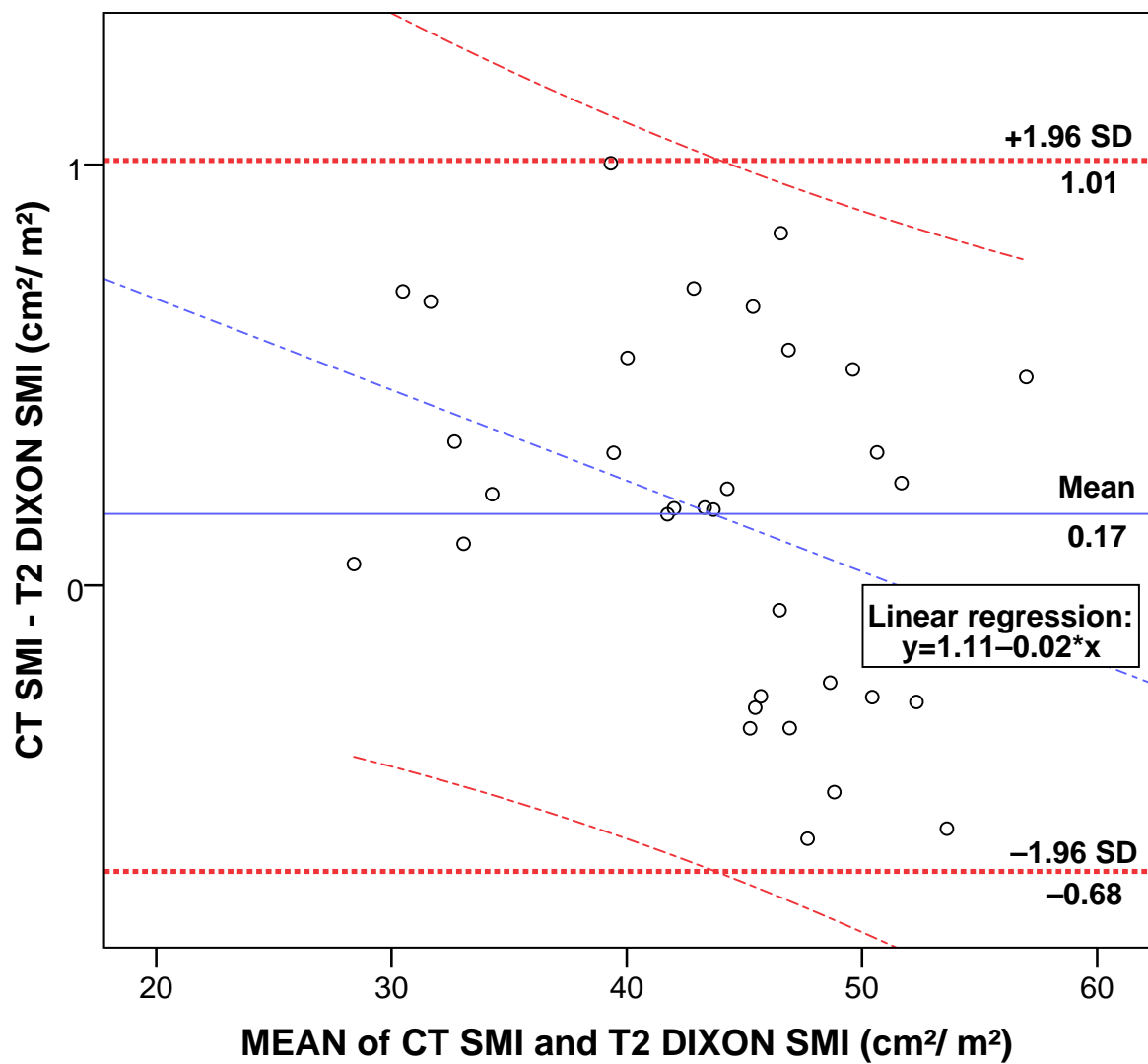

Supplement: Supplementary file 1 — Supporting information Fig. 1. Bland-Altman plots with mean SMI and ΔSMI between CT and 3 Tesla MRI. Boundaries with the 95% confidence interval (±1.96 times the standard deviation) are given for the mean ΔSMI and linear regression analysis. For all patients of the 3 Tesla group (n=33) CT SMI vs. T1 SMI (a), CT SMI vs. T1with contrast SMI (b), CT SMI vs. T1 DIXON SMI (c) and CT SMI vs. T2 DIXON SMI. Abbreviations: SMI Skeletal Muscle Index, CT Computed Tomography, MRI Magnetic Resonance Imaging (PDF 27.2 kb) [file 330_2020_7440_MOESM1_ESM.pdf]
